# Coronary microvascular function is independently associated with left ventricular filling pressure in patients with type 2 diabetes mellitus

**DOI:** 10.1186/s12933-015-0263-7

**Published:** 2015-08-05

**Authors:** Takayuki Kawata, Masao Daimon, Sakiko Miyazaki, Ryoko Ichikawa, Masaki Maruyama, Shuo-Ju Chiang, Chiharu Ito, Fumihiko Sato, Hirotaka Watada, Hiroyuki Daida

**Affiliations:** Department of Cardiology, Juntendo University School of Medicine, 2-1-1, Hongo, Bunkyo-ku, Tokyo, 113-8421 Japan; Department of Metabolism and Endocrinology, Juntendo University School of Medicine, Tokyo, Japan

**Keywords:** Type 2 diabetes mellitus, Diastolic dysfunction, Microvascular disease, Coronary flow reserve

## Abstract

**Background:**

Left ventricular (LV) diastolic dysfunction is known as an early marker of myocardial alterations in patients with diabetes. Because microvascular disease has been regarded as an important cause of heart failure or diastolic dysfunction in diabetic patients, we tested the hypothesis that coronary flow reserve (CFR), which reflects coronary microvascular function, is associated with LV diastolic dysfunction in patients with type 2 diabetes.

**Methods:**

We studied asymptomatic patients with type 2 diabetes but without overt heart failure. Transthoracic Doppler echocardiography was performed that included pulsed tissue Doppler of the mitral annulus and CFR of the left anterior descending artery (induced by adenosine 0.14 mg/kg/min). The ratio of mitral velocity to early diastolic velocity of the mitral annulus (E/e′) was used as a surrogate marker of diastolic function. We also evaluated renal function, lipid profile, parameters of glycemic control and other clinical characteristics to determine their association with E/e′. Patients with LV ejection fraction <50%, atrial fibrillation, valvular disease, regional wall motion abnormality, renal failure (serum creatinine >2.0 mg/dl) or type 1 diabetes were excluded. Patients with a CFR <2.0 were also excluded based on the suspicion of significant coronary artery stenosis.

**Results:**

We included 67 asymptomatic patients with type 2 diabetes and 14 non-diabetic controls in the final study population. In univariate analysis, age, presence of hypertension, LV mass index, estimated glomerular filtration rate and CFR were significantly associated with E/e′. Multivariate analysis indicated that both LV mass index and CFR were independently associated with E/e′. In contrast, there were no significant associations between parameters of glycemic control and E/e′.

**Conclusions:**

CFR was associated with LV filling pressure in patients with type 2 diabetes. This result suggests a possible link between coronary microvascular disease and LV diastolic function in these subjects.

## Background

Epidemiologic studies have shown that patients with diabetes mellitus have a two to fourfold increased risk of cardiovascular mortality [1]. With regard to heart failure, the Framingham study [2] revealed that the risk for congestive heart failure is greatly increased in patients with diabetes, and this is independent of underlying coronary artery disease and other cardiovascular risk factors. Diabetes also directly contributes to the development of left ventricular (LV) hypertrophy, which also predicts cardiac morbidity and mortality [3–5]. Moreover, numerous reports have identified LV diastolic dysfunction using echocardiography as a major early feature of myocardial damage in patients with diabetes [6–8]. Indeed, diastolic parameters are related to prognosis in diabetic patients without overt heart disease [9, 10].

Although the exact causes of LV myocardial damage in patients with diabetes (so called diabetic cardiomyopathy) remain unclear, several factors such as a metabolic abnormality, autonomic dysfunction, myocardial fibrosis and reduced perfusion due to small vessel disease, have been reported as potential mechanisms of myocardial damage in diabetic patients [11, 12]. Small vessel disease that causes diabetic retinopathy represents an increased risk of heart failure that is independent of known risk factors [13], since diabetic retinopathy is significantly associated with LV diastolic dysfunction [14]. Furthermore, there is much less coronary flow reserve (CFR) in diabetic patients with than without retinopathy [15]. CFR, the ratio of hyperemic to basal coronary blood flow velocity, is a physiological parameter that reflects coronary microvascular function in the absence of large vessel stenosis [16], and many reports have described the utility of measuring CFR by noninvasive transthoracic Doppler echocardiography in various diseases including diabetes [17–21]. Therefore, we tested the hypothesis that CFR assessed by transthoracic echocardiography is associated with LV diastolic function in patients with type 2 diabetes without a history of heart failure.

## Methods

### Study subjects and protocol

The present study was a prospective, cross-sectional study. A total of 75 consecutive asymptomatic patients with type 2 diabetes (57 male; mean age, 57 ± 12 years), who were admitted to our institution (Juntendo University Hospital, Tokyo, Japan) for a diabetic educational program were enrolled. In addition, 14 age- and gender-matched non-diabetic subjects were enrolled to serve as a control group. Patients were included if they met the following inclusion criteria: adult type 2 diabetes, no symptoms or history of cardiovascular disease, LV ejection fraction ≥50%, absence of regional LV wall motion abnormalities, and clinically stable. Exclusion criteria were: atrial fibrillation, history of cardiovascular disease, congenital heart disease, significant valvular disease, renal failure (serum creatinine >2.0 mg/dl), and type 1 diabetes. Further, patients with CFR <2.0 or with a positive exercise stress test were also excluded from the study because of the suspicion of significant coronary artery stenosis [22, 23]. We performed coronary flow velocity measurement after conventional echocardiography and venous blood sampling. The study protocol was approved by the Institutional Review Board of Juntendo University Hospital.

### Definition of diabetes and comorbidities

The diagnosis of diabetes was assured in all patients by determination of glucose in the fasting state based on the criteria of the World Health Organization [24]. Dyslipidemia was defined as low-density lipoprotein cholesterol ≥ 140 mg/dl or high-density lipoprotein cholesterol  < 40 mg/dl or triglycerides ≥ 150 mg/dl or already receiving medical treatment. Hypertension was defined as systolic blood pressure >140 mmHg or diastolic blood pressure >90 mmHg or already receiving medical treatment.

### Exercise stress test

A standard double Master’s two-step test was performed to screen patients for significant coronary artery disease [25]. The test endpoint was either chest symptom or completion of the required number of steps within 3 min. A 12-lead electrocardiogram was recorded before, immediately after and at 3, 5, and 7 min after exercise. The test was considered positive if the ST segment (80 ms after the J point) was horizontal or had a downslope with a 1-mm depression.

### Echocardiography

In all subjects, cardiac chamber quantification by 2D echocardiography was performed according to the American Society of Echocardiography guidelines [26]. LV diameters were measured using 2D echocardiography according to the recommended criteria. The thickness of the interventricular septum and the LV posterior wall was measured at end-diastole. LV mass was calculated using diastolic measurements of LV diameter and wall thickness on 2D echocardiography according to the formula recommended by the guidelines. LV end-diastolic volume and end-systolic volume were determined from the apical views using modified biplane Simpson’s method. LV ejection fraction was calculated by the following equation: 100 × (end-diastolic volume − end-systolic volume)/end-diastolic volume. Each parameter was indexed for body surface area, when appropriate. For assessing conventional diastolic parameters, mitral inflow velocities were determined by pulsed Doppler imaging. The peak early (E) and late (A) diastolic velocity, the deceleration time (DcT) from the peak of the early diastolic wave to baseline and the E/A ratio were assessed from the mitral inflow velocity pattern. The mitral annular motion velocity was recorded at the septal mitral annulus site in the apical 4-chamber view using pulsed tissue Doppler. Peak early diastolic velocity (e′) of the annulus was measured, and the ratio of peak early diastolic transmitral flow velocity to annular velocity (E/e′) was calculated. Since E/e′ has been accepted as a surrogate marker of LV filling pressure [27], particularly in heart failure with preserved ejection fraction, we used E/e′ as an index of diastolic function in this study. Furthermore, according to previous reports [27, 28], we classified diastolic function in all study subjects into one of four categories (normal, grade I, grade II or grade III) to compare the prevalence of diastolic dysfunction between controls and diabetic patients.

### Laboratory measurements

Venous blood samples for determination of serum creatinine, fasting blood sugar, glycosylated hemoglobin, immunoreactive insulin and lipid profiles (triglycerides, high-density lipoprotein cholesterol and low-density lipoprotein cholesterol) were drawn in all patients immediately before the CFR measurement. Moreover, the homeostasis model assessment ratio (HOMA-R) index was calculated as fasting plasma glucose × fasting plasma insulin/405 for assessing insulin resistance [29]. Estimated glomerular filtration rate (eGFR) was determined based on the new Japanese coefficient-modified Modification of Diet in Renal Disease (MDRD) study equation [30]. The formula is as follows: eGFR = 194 × serum creatinine (SCr)^−1.094^ × age-0.287, where age is in years, SCr is in mg/dL and GFR is in mL/min/1.73 m^2^ body surface area. The product of this equation was multiplied by a correction factor of 0.739 in women.

### Measurement of CFR

**Fig. 1:**
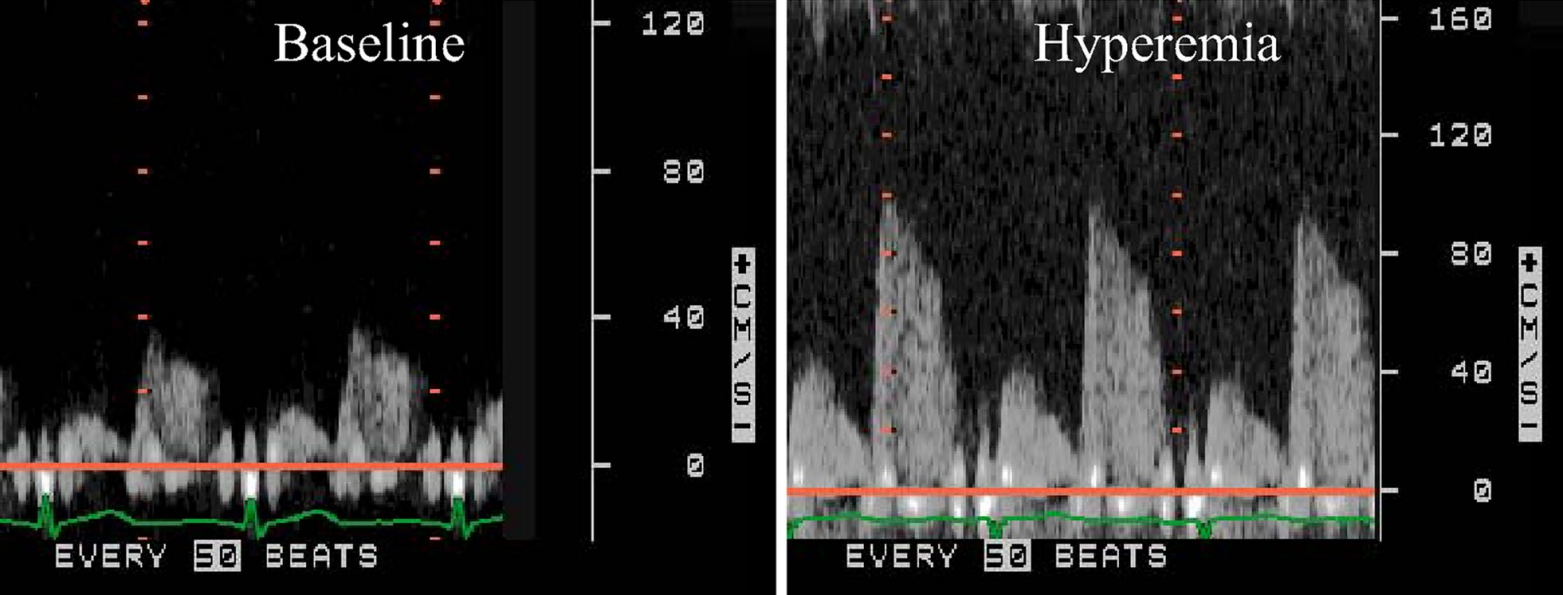
Example of Doppler tracing of LAD blood flow during baseline and after adenosine injection (hyperemia).

Blood flow velocity in the distal portion of the left anterior descending (LAD) coronary artery was measured with a high-frequency transducer (5–7 MHz) under color Doppler flow guidance. With a sample volume (2.5 or 3.0 mm wide) positioned on the color signal in the LAD, Doppler spectral tracings of flow velocity in the LAD were recorded using a fast Fourier transformation. We first recorded baseline spectral Doppler signals in the LAD, then an intravenous adenosine was administered (0.14 mg/kg/min) to record spectral Doppler signals during hyperemia (Fig. 1). Mean and peak diastolic velocities were measured at baseline and peak hyperemic conditions from the Doppler signal recordings. Measurements were averaged over three cardiac cycles. In this study, CFR was defined as the ratio of hyperemic to basal mean diastolic coronary flow velocity. To determine the reproducibility of mean diastolic velocity, a total of ten randomly selected measurements were analyzed twice by one investigator at a 1-week interval and once by another investigator.

### Statistical analysis

All data are expressed as mean ± standard deviation (SD) or the number (percent) of patients. Data in the diabetes patients and controls were compared using unpaired *t* test for continuous variables and Chi square test for categorical variables. In patients with diabetes, univariate regression analysis was performed to determine the association between E/e′ and the following variables: age, gender, body mass index, presence of hypertension, rate pressure product (systolic blood pressure × heart rate), LV ejection fraction, LV mass index, eGFR, fasting blood sugar, glycosylated hemoglobin, HOMA-R, lipid profile and CFR. The variables that were significant in the univariate model were then entered into a multivariate regression analysis using a forward stepwise method. A probability value of p < 0.05 was considered significant. All data were statistically analyzed using JMP version 8.0 (SAS Institute, Cary, NC, USA).

## Results

### Enrolled patients

**Fig. 2:**
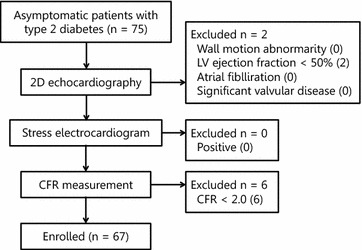
Patient flow diagram. Of the consecutive 75 patients who were studied, 8 were excluded. Sixty-seven patients were enrolled and completed the entire protocol.

Among 75 patients, 2 patients (3%) were excluded because they had LV ejection fraction <50%. None of the patients had a positive exercise stress test. Six patients (8%) had CFR <2.0. Therefore, the final study population consisted of 67 patients (50 male; mean age, 57 ± 12 years) with type 2 diabetes who met the inclusion criteria (Fig. 2). All 14 of the non-diabetic controls had CFR ≥2.0, LV ejection fraction ≥50%, no symptoms or history of cardiovascular disease, and no regional LV wall motion abnormalities.

### Patient characteristics and echocardiographic data

**Fig. 3:**
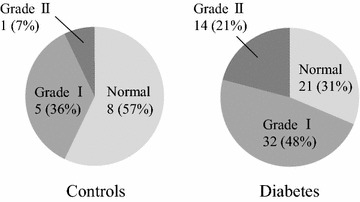
Distribution of diastolic dysfunction in age-matched controls and patients with type 2 diabetes.

Patients’ general characteristics, laboratory and echocardiographic data are summarized in Tables 1 and 2. Seventy-four % of our study subjects were male. Although controls of blood pressure and lipid profile were adequate, 57% had hypertension and 51% had dyslipidemia. About half of the patients were on inhibitors of the renin-angiotensin system or statins. With respect to diabetes control, 51% of patients were receiving insulin by subcutaneous infusion. Patients with diabetes had a dilated left atrium, elevated E/e′ and impaired CFR compared with controls; however, the other characteristics and echocardiographic parameters were not different between the two groups. Figure 3 shows the distribution of diastolic dysfunction grade in diabetic patients and controls. Most of the patients (69%) had diastolic dysfunction, indicating a higher prevalence of diastolic dysfunction in the diabetic population. None of the subjects had grade III diastolic dysfunction.

**Table 1:** General characteristics of the study population

Variables	Patients (n = 67)	Controls (n = 14)	p
Age (years)	57 ± 12	56 ± 10	0.74
Male (%)	50 (74)	9 (64)	0.31
BMI (kg/m^2^)	25.8 ± 4.6	24.4 ± 3.7	0.24
SBP (mmHg)	118 ± 17	124 ± 12	0.13
DBP (mmHg)	63 ± 12	68 ± 7	0.075
HR (bpm)	68 ± 11	68 ± 8	0.94
RPP	8034 ± 1713	8558 ± 1233	0.26
Hypertension (%)	38 (57)	9 (64)	0.42
Dyslipidemia (%)	34 (51)	8 (57)	0.44
Diabetes treatment			
Diet (%)	10 (15)	–	
Sulfonylurea (%)	11 (16)	–	
Glinide (%)	9 (13)	–	
Biguanide (%)	22 (33)	–	
Thiazolidinedione (%)	9 (13)	–	
α-glucosidase inhibitor (%)	23 (34)	–	
Insulin (%)	34 (51)	–	
Other treatment			
Statin (%)	30 (45)	5 (36)	0.38
ACE inhibitor/ARB (%)	34 (51)	5 (36)	0.23
CCB (%)	23 (34)	7 (50)	0.21

Data are presented as number (%) or mean ± SD.

*BMI* body mass index, *SBP/DBP* systolic/diastolic blood pressure, *HR* heart rate, *RPP* rate pressure product, *ACE inhibitor/ARB* angiotensin-converting enzyme inhibitor/angiotensin-receptor blocker, *CCB* calcium channel blocker.

**Table 2:** Laboratory and echocardiographic data

Variables	Patients n = 67	Controls n = 14	p
Laboratory data			
FBS (mg/dl)	130.9 ± 40.1	97.0 ± 6.7	<0.0001
HbA1c (%)	7.8 ± 1.5	5.2 ± 0.2	<0.0001
IRI (µU/l)	8.8 ± 8.3	–	
HOMA-R	2.9 ± 3.2	–	
TG (mg/dl)	112.7 ± 50.2	120.0 ± 48.1	0.64
HDL-C (mg/dl)	46.1 ± 14.0	48.3 ± 7.7	0.43
LDL-C (mg/dl)	107.5 ± 27.2	114.0 ± 31.9	0.50
Creatinine (mg/dl)	0.79 ± 0.19	0.74 ± 0.14	0.31
eGFR ml/min./1.73 m^2^	78.1 ± 19.3	79.9 ± 13.3	0.69
Echocardiographic data			
Left atrial diameter (mm)	36.2 ± 4.4	33.5 ± 4.3	0.044
RWT	0.43 ± 0.07	0.43 ± 0.06	0.72
LV mass index (g/m^2^)	84.7 ± 21.4	77.1 ± 14.7	0.13
LVEDV index (ml/m^2^)	55.8 ± 11.4	54.3 ± 8.9	0.61
LVESV index (ml/m^2^)	18.2 ± 5.0	18.5 ± 3.3	0.81
LVEF (%)	67.2 ± 5.1	65.6 ± 5.1	0.34
E/A	0.9 ± 0.3	0.9 ± 0.2	0.42
DT (msec)	194.8 ± 45.1	182.9 ± 42.0	0.37
Septal e′ (cm/sec)	7.0 ± 2.2	7.9 ± 1.7	0.11
E/e′	9.3 ± 2.7	7.6 ± 1.1	0.0004
MDV baseline (cm/sec)	15.3 ± 3.5	15.2 ± 3.9	0.89
MDV hyperemia (cm/sec)	50.6 ± 12.6	56.8 ± 11.6	0.088
CFR	3.3 ± 0.8	3.8 ± 0.7	0.036

Data are presented as mean ± SD.

*FBS* fasting blood sugar, *HbA1c* glycated hemoglobin, *IRI* immunoreactive insulin, *HOMA-R* homeostasis model assessment ratio, *T-Cho* total-cholesterol, *HDL-C* high-density lipoprotein cholesterol, *LDL-C* low-density lipoprotein cholesterol, *eGFR* estimated glomerular filtration rate, *RWT* relative wall thickness, *LVEDV/ESV* left ventricular end-diastolic volume/end-systolic volume, *LVEF* left ventricular ejection fraction, *DT* deceleration time of mitral E wave, *MDV* mean diastolic coronary flow velocity, *CFR* coronary flow reserve.

### CFR and clinical variables

Both age and RPP were significantly associated with CFR (age, β = −0.33, p = 0.0056; RPP, β = −0.31, p = 0.0095). There was a tendency for eGFR to be associated with CFR (β = 0.23, p = 0.057). There was no association between temporal glycemic control and CFR (fasting blood sugar, β = −0.11, p = 0.34; glycosylated hemoglobin, β = 0.053, p = 0.67; HOMA-R, β = −0.014, p = 0.39). Moreover, there were also no association between lipid profile and CFR (LDL-C, β = 0.10, p = 0.38; HDL-C, β = −0.014, p = 0.91; TG, β = −0.12, p = 0.31). CFR measured from peak diastolic velocity in diabetic patients was 3.3 ± 0.7. There was a strong correlation between CFR measured using mean diastolic flow velocity and CFR measured using peak diastolic flow velocity (r = 0.92, p < 0.0001). The inter- and intra-observer variabilities for the measurement of coronary Doppler velocity were 5.0 and 3.9%, respectively.

### Univariate and multivariate determinants of E/e′

**Fig. 4:**
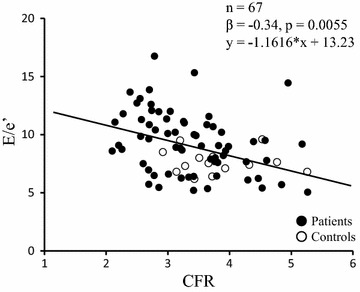
Inverse association between CFR and surrogate marker of LV filling pressure (E/e′) in patients with type 2 diabetes.

Univariate and multivariate potential determinants of E/e′ were analyzed and summarized in Table 3. In univariate analysis, age, presence of hypertension, LV mass index, eGFR and CFR were significantly associated with E/e′. Female gender tended to show an association with high E/e′. Figure 4 shows the relation between CFR and E/e′; a significant inverse association between CFR and E/e′ was observed. CFR was also associated with e′, an index of myocardial relaxation (β = 0.26, p = 0.0319). There were no significant associations between E/e′ and parameters of glycemic control such as fasting blood sugar, glycosylated hemoglobin or HOMA-R. Multivariate analysis (including age, presence of hypertension, LV mass index, eGFR and CFR) showed that both LV mass index and CFR were independent determinants of E/e′ (Table 3).

**Table 3:** Univariate and multivariate analysis of the variables associated with E/e′ (n = 67)

Variables	Univariate		Multivariate	
	β	p	β	p
Age	0.36	0.0033	0.22	0.12
Female	0.21	0.086		
BMI	−0.048	0.70		
Hypertension	0.32	0.0095	0.13	0.29
RPP	−0.035	0.78		
LVEF	0.17	0.17		
LV mass index	0.33	0.0076	0.27	0.023
eGFR	−0.25	0.042	0.00025	0.99
FBS	0.11	0.40		
HbA1c	−0.079	0.53		
HOMA-R	0.016	0.89		
LDL-C	0.00056	0.99		
TG	0.084	0.50		
CFR	−0.34	0.0055	−0.24	0.043

Abbreviations as in Tables 1 and 2.

## Discussion

The key finding of the present study was that CFR was independently and inversely associated with E/e′, a surrogate marker of LV filling pressure, in patients with type 2 diabetes without overt cardiovascular disease.

Earlier studies have demonstrated that microalbuminuria caused by diabetic nephropathy is strongly associated with cardiovascular disease and mortality [31]. Moreover, retinopathy has been reported to be associated with congestive heart failure or LV diastolic dysfunction in diabetic patients [13, 14]. Based on these findings, microvascular abnormalities have been regarded as an important cause of heart failure in diabetic patients. However, there are limited data on the direct relationship of coronary microvascular function with LV diastolic function in patients with diabetes. Multivariate analysis in the present study showed that both LV mass index and CFR were independently associated with E/e′ in type 2 diabetic patients without overt cardiovascular disease. This result suggests that coronary microvascular disease is one of the determinants of LV diastolic dysfunction, recognized as an early change in diabetic cardiomyopathy. However, it is also possible that the LV diastolic dysfunction may have affected maximum coronary flow and CFR. Although our results fill an important gap in the earlier published data, the association between coronary microvascular function and LV diastolic dysfunction does not prove that coronary microvascular function is the cause of LV diastolic dysfunction in patients with diabetes.

In the present study, CFR was significantly lower in diabetic patients than controls, and this result agrees with the previous studies. It is conceivable that the reduction of CFR in type 2 diabetes is a complex phenomenon that is related to several factors. Diabetic patients are known to have structural abnormalities in small vessels, including a reduced capillary volume, fibrosis in vessel walls, and thickening of capillary basement membranes [32]. Although an experimental study indicated that endothelium-dependent dilatation of the coronary microvasculature at the early phase of diabetes is preserved despite increased oxidative stress [33], endothelial dysfunction due to oxidative stress, accumulated glycosylation end products, or activated protein kinase C may partially explain the reduced CFR observed in diabetic patients [32].

We used adenosine to assess CFR in this study. Because adenosine dilates coronary arterioles through adenosine A2 receptors by increasing adenylate cyclase and decreasing calcium uptake [34], the blood flow response to adenosine is thought to be endothelium-independent. However, it has been reported that adenosine also acts, at least partially, as an endothelium-dependent vasodilator, both via flow-mediated dilation and via direct stimulation of endothelial cells [35, 36]. We also reported previously that asymmetric dimethylarginine, an endogenous nitric oxide synthase inhibitor, was inversely associated with CFR in diabetic patients [37]. Therefore, adenosine-induced CFR partially reflects coronary endothelial function [36].

Recently, a new paradigm [38] for heart failure with preserved ejection fraction has been proposed; a systemic proinflammatory state due to diabetes and comorbidities causes coronary microvascular endothelial inflammation, and this reduces nitric oxide bioavailability, cyclic guanosine monophosphate content, and protein kinase G activity in adjacent cardiomyocytes. Furthermore, low protein kinase G activity favors muscle fiber hypertrophy development and increases resting tension because of hypophosphorylation of titin, and both stiff cardiomyocytes and interstitial fibrosis contribute to the development of high diastolic LV stiffness and heart failure. This novel paradigm supports our findings, and the measurement of CFR by echocardiography may be a noninvasive practical tool to assess coronary endothelial function in the clinical setting.

Contrary to previous reports [39], we failed to find a correlation between E/e′ and control parameters of diabetes such as fasting blood sugar, glycosylated hemoglobin, immunoreactive insulin and HOMA-R in the present study. This result may be because of the small number of patients in the present study, differences in patients’ clinical backgrounds and/or the effect of treatment. However, in subjects with albuminuria and normal LV ejection fraction, the HOPE study [40] indicated that heart failure risk greatly rises in patients with a high degree of albuminuria irrespective of the presence or absence of diabetes. This result suggests that the degree of microvascular disease due to diabetes or some other factor is associated with heart failure events rather than indices of glycemic control per se. Therefore, it is not surprising that there was no relationship between temporal glycemic control and E/e′ in the present study. As for CFR, there was also no association between temporal glycemic control and CFR in the present study. As with albuminuria, CFR may be a summary measure of microvascular injury due to multiple risk factors including diabetes. Furthermore, CFR is also associated with LV diastolic dysfunction in patients with arterial hypertension [41] and coronary artery disease [42], and an association between CFR and LV diastolic function may also occur in patients with various coronary risk factors.

### Limitations

There are several important limitations of the present study. The presence of epicardial coronary stenosis was excluded based only on the absence of symptoms, no previous history of cardiovascular disease, and a negative exercise stress test. Advanced imaging modalities (e.g. stress myocardial perfusion scintigraphy, computed tomographic angiography, conventional coronary angiography) were not applied. Therefore, we cannot be fully confident that we excluded patients with epicardial coronary stenosis. However, all of the studied patients had both a negative exercise stress test and CFR ≥2.0, indicating that there was no significant LAD stenosis [22, 23]. It has also been reported that CFR <2.0 by transthoracic Doppler echocardiography has a high predictive value for the presence of significant LAD stenosis, even in a population that includes patients with diabetes [43]. Therefore, we assumed that there was little likelihood of including patients with significant LAD stenosis in our study population. However, the exclusion of patients with CFR <2.0 may have excluded some patients with severe microvascular dysfunction, and this is an important study limitation. Moreover, CFR was measured only in the LAD. Although measurement of CFR in three coronary arteries is preferable, this remains technically challenging at the present time. However, coronary microcirculatory dysfunction affects the left ventricle globally as well as regionally [44]; therefore, CFR assessment in the LAD is an excellent option for evaluating the global coronary microcirculation. Although diabetic patients had significantly lower CFR than controls, CFR in diabetics in this study was 3.3 and relatively high. Even if the 6 patients with CFR <2.0 were included, the mean value of CFR was still 3.1. The CFR value in similar subjects in our previous reports was 2.9 [17, 37]. This may be because of various differences in patients’ background. For example, only 5 patients had microalbuminuria (>30 mg/g creatinine) in this study, and the microvascular damage in diabetic patients in the present study may not have been as severe as in our previous studies. Although E/e′ is now widely recognized as a surrogate marker of LV filling pressure, particularly in patients with preserved LV ejection fraction, LV filling pressure was not confirmed by invasive methods in the present study.

## Conclusion

Coronary microvascular function was independently associated with LV filling pressure in patients with type 2 diabetes but without overt cardiovascular disease. This result suggests a possible link between coronary microvascular disease and LV diastolic function in these subjects.
